# A nosocomial outbreak of *Ralstonia mannitolilytica* linked to cosmetic water mist sprays in the intensive care unit of a tertiary care hospital, Switzerland, 2024

**DOI:** 10.2807/1560-7917.ES.2025.30.49.2500287

**Published:** 2025-12-11

**Authors:** Alicia Cancela Costa, Dominique S Blanc, Claire Bertelli, Trestan Pillonel, Damien Jacot, Léa Griess, Jean-Luc Pagani, Bruno Grandbastien, Rami Sommerstein, Marie Nahimana Tessemo, Laurence Senn, Estelle Moulin

**Affiliations:** 1 Infection Prevention and Control Unit, Infectious Diseases Service, Lausanne University Hospital and University of Lausanne, Lausanne, Switzerland; 2 Institute of Microbiology, Lausanne University Hospital and University of Lausanne, Lausanne, Switzerland; 3 Intensive Care Unit, Lausanne University Hospital and University of Lausanne, Lausanne, Switzerland; 4 Faculty of Health Sciences and Medicine, University of Lucerne, Lucerne, Switzerland; 5 Department of Infectious Diseases, Bern University Hospital and University of Bern, Bern, Switzerland; 6 Cantonal Unit for Hygiene, Prevention, and Infection Control Vaud; Cantonal Doctor Office, Public Health Department, Canton of Vaud, Lausanne, Switzerland; * These authors contributed equally to this work and share last authorship.

**Keywords:** Nosocomial outbreak, Ralstonia, water spray

## Abstract

*Ralstonia mannitolilytica* is a rare emerging, multidrug-resistant, opportunistic pathogen known to cause nosocomial outbreaks associated with hospital water sources and medical devices. In June 2024, the microbiology laboratory of Lausanne University Hospital alerted the Infection Prevention and Control Unit following the detection of *R. mannitolilytica* in clinical samples from two intensive care unit (ICU) patients. This triggered a comprehensive epidemiological investigation, including extensive environmental sampling and whole genome sequencing of the isolates. Between May and June 2024, *R. mannitolilytica* was detected in three ICU patients. Activation of the Swissnoso network led to the identification of an additional case in a rehabilitation centre. Environmental investigations traced the source to commercial cosmetic water mist sprays used for patient care. The sequences confirmed a genetic match between patient and spray isolates, prompting the immediate withdrawal of this product. This nosocomial outbreak of *R. mannitolilytica* revealed an unexpected and seemingly innocuous source of contamination – water mist sprays – highlighting the importance of considering cosmetics products used in patient care and questioning their use in patients with risk factors, such as those in ICU or with immunosuppression.

**Key public health message**

**What did you want to address in this study and why?**

We investigated an outbreak of *Ralstonia mannitolilytica* in a Swiss hospital to identify the source and prevent further spread. As *Ralstonia* is a waterborne bacterium, targeted environmental investigations were conducted.

**What have we learnt from this study?**

Unexpectedly, the common source was contaminated water sprays, highlighting the potential risk of cosmetics in hospital settings, particularly in immunosuppressed patients or those in intensive care. Cosmetic products used for patient care could represent a risk for healthcare-associated infections. Extensive environmental sampling and effective communication were crucial to controlling the outbreak.

**What are the implications of your findings for public health?**

It is important to maintain control over non-medical devices used in healthcare settings, particularly for immunocompromised patients. Rapid response systems and national collaboration facilitate the management of outbreaks.

## Background

*Ralstonia mannitolilytica* is a Gram-negative non-fermentative bacterium commonly found in water and soil. It has been implicated in hospital outbreaks due to its ability to resist and survive extreme environmental conditions, particularly in aqueous sources. Naturally resistant to multiple classes of antibiotics, its mode of transmission remains poorly understood. The number of reported cases of infection and nosocomial transmission involving *R. mannitolilytica* (formally *R. pickettii*) and, less frequently, *R. insidiosa*, has been steadily increasing worldwide over the past few decades [1-3]. This rise is partly attributable to advances in microbiological identification methods such as matrix-assisted laser desorption/ionisation time of flight (MALDI-TOF) mass spectrometry and genome sequencing. Previous outbreaks have been reported among immunocompromised patients, including those undergoing oncological or haematological treatments [4-7], haemodialysis patients [8-10], and patients in intensive care units (ICU), neonatal ICU or paediatric ICU [11-19]. Most patients developed bacteraemia, with occasional cases of sepsis and rare deaths. Common sources of contamination included aqueous environments such as dialysis water [8], sterile or distilled water [10,18,20], or saline solutions [7,12,21-24]. Medical devices that could serve as sources of contamination included respiratory equipment [19,25] and dialysis equipment [9,26]. Finally, several outbreaks were linked to contaminated pharmaceutical products administered intravenously, such as heparin [11,14,27] or fentanyl [13,15].

## Outbreak detection

In early June 2024, the microbiology laboratory of the Lausanne University Hospital informed the infection prevention and control (IPC) unit about the detection of *R. mannitolilytica* in clinical samples (blood culture and respiratory sample) collected 1 week apart from two distinct patients. These patients were hospitalised in a 14-bed adult medical and surgical ICU. A third case was incidentally identified in the same unit 2 weeks later during routine weekly screening for extended-spectrum β-lactamase (ESBL) and carbapenemase-producing organisms using a rectal swab. This led to a thorough epidemiological investigation with the aim to identify the common source. We also detail the IPC measures implemented, including decisions regarding the widespread use of the implicated product in healthcare settings.

## Method

### Patient identification and case definition

A case was defined as a patient with *R. mannitolilytica* identified by culture in a clinical sample or from a rectal swab, the latter being part of the routine screening for extended-spectrum beta-lactamase-expressing organisms performed weekly on all ICU patients. Patient data were collected from computerised medical records.

### Environmental investigation

To identify a potential common source of contamination, the IPC team, in collaboration with the ICU head nurses and the equipment manager, conducted sequential environmental sampling for *R. mannitolilytica*. The investigation focused on shared devices, medications and aqueous sources used by the three affected patients. Surface samples were collected using eSwab devices (Copan, Italy), while water from the mist spray product was obtained by discharging 10–15 mL into a sterile plastic bag and transferring it into a sterile 50 mL tube. All procedures were carried out under a Class II biosafety cabinet.

### Bacteriology and whole genome sequencing

As we observed that the clinical isolates of *R. mannitolilytica* grew rapidly on Columbia blood agar incubated at 37 °C for 48 h, we used these conditions to analyse environmental samples. Swabs were streaked on the agar media, whereas the water samples (200 µL) were spread on the surface of the agar media. In addition, as microbiological quality control of purified water according to the European Pharmacopea [28] is done with the R2A medium incubated at 20 °C for 72 h, we also used these conditions in parallel. Identification of isolated colonies was performed with MALDI-TOF (Bruker, Germany). We subjected the *R. mannitolilytica* isolates from patients and environmental samplings to genomic typing. Genomic DNA was extracted with a QIACube Connect MDX using the QIAamp DNA Mini Kit (Qiagen, Germany). Libraries were prepared using the DNA Prep library preparation kit. Sequencing was performed on an Illumina MiSeq platform (Illumina, United States), generating 151 bp or 251 bp paired-end reads. Reads were assembled using an in-house Snakemake workflow (https://github.com/metagenlab/diag_pipelines, version 2.8.3). The genome of the isolate from Patient 1 was used as a reference. 

## Results

### Patient characteristics

#### Patient 1

The first *R. mannitolilytica* isolate was identified at the end of May 2024, in a bronchial aspirate from a patient who had been hospitalised for several weeks in the ICU following a transplant. The patient also underwent multiple abdominal surgeries because of digestive complications.

#### Patient 2

The second *R. mannitolilytica* isolate was identified at the beginning of June 2024, in a blood culture taken from a central catheter from a patient who had been hospitalised for several weeks in the same ICU following a cardiac valve replacement for valvulopathy. Peripheral blood cultures were sterile; follow-up blood cultures after catheter removal, without targeted antibiotic therapy, and the catheter culture were also sterile. It was therefore not considered a central-line associated bloodstream infection. Subsequent bronchial aspirates taken the following weeks were positive for *R. mannitolilytica*.

#### Patient 3

The third isolate was identified mid-June 2024, from a routine rectal swab taken from a patient hospitalised for several weeks in the same ICU for multiple complications of a carcinoma.

In all three patients, *R. mannitolilytica* was considered a coloniser (respiratory, central venous catheter and digestive colonisation, respectively) and did not cause any infection. All patients had complex, multi-morbid conditions and had undergone multiple surgical procedures as well as endoscopic examinations. No deaths were attributable to *R. mannitolilytica* infection.

### Environmental samples

The environmental investigation was initiated after the identification of the third case. We collected 86 environmental samples in the ICU and in the operating theatre between 19 June and 15 July 2024 (Table 1). Samples were taken from various medical devices, medical products, medicines and cosmetics, including the extracorporeal membrane oxygenation (ECMO) machine (n = 7), the mechanical ventilation humidifier cascade (n = 14), the dialysis machine (n = 1), and the body temperature control system (n = 5) (Table 1). Given the ability of *Ralstonia* spp. to survive in an aqueous environment, we also sampled the drains traps in patient rooms and waste disposal areas.

**Table 1:** Results of cultures for *Ralstonia mannitolilytica* from samples collected in the environment of colonised patients, Switzerland, 2024 (n = 86)

Date	Item		Number of samples	Results
19–20 June	ECMO device (connector, water tank, water from tank, filtered water, water after double disinfection), drain traps tap water in the operating theatre		14	Negative
25 June	Body cleansing wipes (2% chlorhexidine wipes, wipes without chlorhexidine), liquid hand soap		5	Negative
27 June	Drain traps in patient rooms and waste disposal area		9	Negative
1 July	Medical products and cosmetics (ultrasound gel, sterile water for injection, sterile water for oxygen humidification, moisturising), liquid hand soap		11	Negative
2 July	Mechanical ventilation humidifier cascade in a positive patient	Water in cascade	1	*R. mannitolilytica*
		Patient-side location	5	*R. mannitolilytica*
		Machine-side locations and water from heater plate	8	Negative
2 July	Dialysate fluid		1	Negative
2 July	Patient temperature control system		5	Negative
11 July	Drinking water (fountain, bottles)		2	Negative
11 July	Medicines (iv furosemid, glucosalin, glucosalin, heparin vial, aqua ad injectabilia, NaCl 0.9%, prefilled NaCl 0.9% syringe), chlorhexidine solution 0.2%, alcoholic solution for skin disinfection, ultrasound gel, cosmetics (shaving foam, shampoo, shampoo cap, dental adhesive cream, glycerol mouth care sticks, skin protective film, artificial saliva spray, toothpaste)		21	Negative
11–15 July	Water mist sprays		2	*R. mannitolilytica*
			2	Negative

ECMO: extracorporeal membrane oxygenation; iv: intravenous.

We first found *R. mannitolilytica* on various components of the mechanical ventilation humidifier cascade used by one of the contaminated patients. However, all positive samples were obtained from the patient's side (particularly from the tracheostomy and expiratory section), while upstream samples, including those from the tank, were sterile. This led to the conclusion that the device was contaminated by the patient, rather than the cascade being the source.

Following negative results from initial environmental sampling, the investigation was expanded on 11 July to include testing of fountain water, injectable medications and cosmetic products (Table 1). We subsequently detected *R. mannitolilytica* in a commercial water mist spray with propellant gas, which had been used for patient refreshment and oral care. A thorough investigation of this product was undertaken, including the analysis of different batches that had been recalled to the hospital purchasing centre which supplies several hospitals in the region. Among the 13 batches tested, four were found to be contaminated (Table 2). Interestingly, the use of Columbia agar incubated at 37 °C resulted in a higher number of positive batches and increased bacterial counts as compared with growth conditions for purified water testing (R2A, 20°C). All three affected patients had been exposed to these contaminated water mist sprays, as well as a fourth patient identified in another hospital (see below).

**Table 2:** Quantitative bacteriological results (cfu/mL) for different batches of a commercial water spray, Switzerland, 2024 (n = 13)

Batch	Number of vials analysed	Columbia 37 °C 48 h		R2A 20 °C 72 h	
		*Ralstonia mannitolilytica*	Others^a^	*Ralstonia mannitolilytica*	Others^a^
2022 10 09	2	0	0	0	0
2024 03 31	2	0	18–121	0	40–170
2025 10 26	2	0	0	0	0
2025 11 02	2	0	0–45	0	0–32
2025 11 03	4	0	0–14	0	0–21
2025 11 06	2	0	0 to >200	0	0 to >200
2026 02 12	2	0	0	0	0
2026 02 19	2	0	0	0	0
2026 03 13	2	0	0	0	0
2026 10 02	4	1	85 to >200	0	16 to >200
2026 10 03	5	0–31	0	0	11–100
2026 10 04	6	1–57	0–1	0–10	0 to >200
2026 10 05	2	0–1	5–7	0	2–6

Two culture conditions were tested: one mirroring clinical samples (Columbia agar, 37 °C) and one following the European Pharmacopoeia method for purified water (R2A agar, 20 °C).

^a^ Other species identified: *Brevundimonas aurantiaca, Sphingomonas spp., Sphingomonas melonis, Methylobacterium radiotolerans*.

### Genomic typing

**Figure 1:**
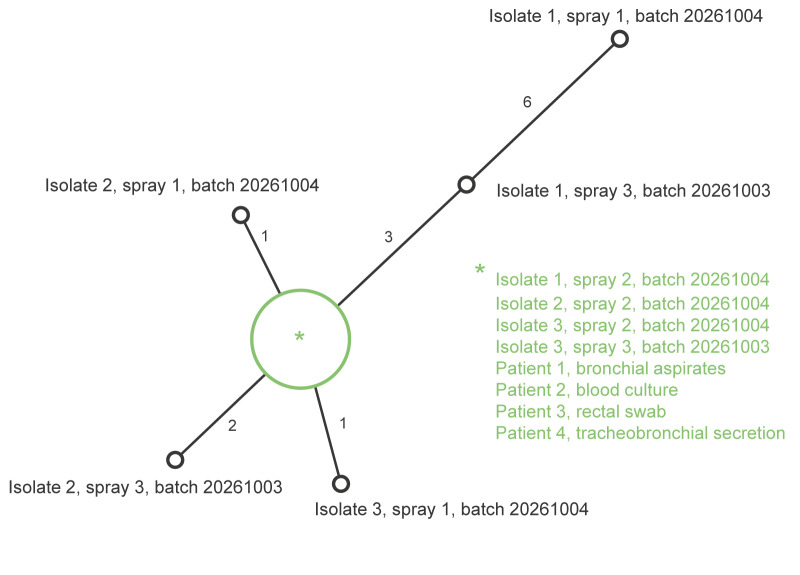
Minimum spanning tree of *Ralstonia mannitolilytica* genomes, Switzerland, 2024 (n = 13)

Genome comparison of 13 sequenced *R. mannitolilytica* isolates, including four from patients and nine from three distinct water mist sprays from two different batches, revealed that all isolates were genetically very similar (Figure 1) [29]. Four patient isolates and four water spray isolates were strictly identical, highly suggesting that the water sprays were the source of bacterial contamination. The remaining five water spray isolates exhibited between one and seven polymorphisms compared with the patient isolates across the entire genome, indicating some genetic diversity within the *R. mannitolilytica* population present in the sprays.

Circles group genetically identical isolates; numbers indicate whole genome single nucleotide polymorphism differences between isolates.

## Outbreak control measures

Contact precautions were implemented for all affected patients. In addition, measures were taken to strengthen the application of standard precautions and compliance with contact isolation, including reminders to the medical and the nursing team, seminars and supervision by the IPC team.

**Figure 2:**
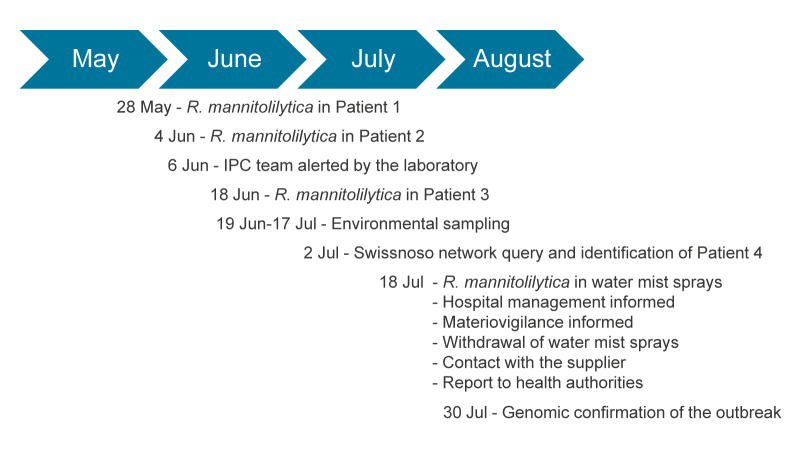
Timeline summarising the key events of the *Ralstonia mannitolilytica* hospital outbreak and investigations, Switzerland, 2024

Upon receiving the results demonstrating the presence of *R. mannitolilytica* in the water sprays, the hospital's Chief Executive Officer and the medical device vigilance officer were notified. They subsequently ordered the immediate withdrawal and quarantine of all water sprays, irrespective of batch or brand, throughout the entire institution on 18 July (Figure 2).

IPC: infection prevention and control.

Simultaneously, the information was reported to the Cantonal Medical Office, the national centre of infection control Swissnoso, the Federal Office of Public Health (FOPH) and the Federal Food Safety and Veterinary Office (FSVO). Through the network of Swissnoso members, another case was identified in a rehabilitation centre (Patient 4) who tested positive for *R. mannitolilytica* in a tracheobronchial secretion and was also considered a colonisation. Genotypic analysis revealed that this isolate was indistinguishable from isolates detected in our hospital. Moreover, the same type of sprays, specifically from the same batch as the contaminated ones, were used for patient oral care of intubated patients at this institution. Unfortunately, these sprays were no longer available for analysis, preventing confirmation of a common contamination source.

In our hospital, a final decision was made to discontinue the use of water sprays, regardless of brand, as well as any related products, for patient care within the institution. To date (November 2025), *R. mannitolilytica* has never been detected again in any clinical sample in our hospital.

## Discussion

We report a nosocomial outbreak of *R. mannitolilytica* associated with contaminated commercial water mist sprays with propellant gas, which resulted in the decision to discontinue the use of this type of cosmetic product in our healthcare institution.

Outbreaks of *Ralstonia* spp. linked to contaminated fluids or medical devices have been reported, but none involving cosmetics. In contrast to previous outbreaks, where blood cultures were typically positive, only one patient presented with a positive blood culture, while the remaining samples were bronchial aspirates and a rectal swab. Contamination of an injectable product was therefore considered unlikely, prompting us to focus on aqueous devices and products in contact with the airways.

It is noteworthy that water mist sprays are regulated as cosmetic products, and not as medical devices. The Swiss agency for therapeutic products (Swissmedic) defines a medical device as any instrument, apparatus, appliance, material or other article intended for use on human beings for the diagnosis, prevention, monitoring or alleviation of disease [30]. Consequently, in Switzerland, water sprays fall under the regulations of the FSVO instead of Swissmedic. They are thus governed by a different legal framework and set of standards. As a result, their manufacturing standards are designed to meet the requirements for the general population in good health. They usually contain pasteurised water, and each batch undergoes microbiological testing before being released to the market. However, this process does not eliminate the presence of opportunistic pathogens, particularly for at-risk patients. In Switzerland, the microbiological safety of cosmetic products is governed by the Ordinance on Cosmetics (OCos), which is aligned with the European Regulation (EC) Number 1223/2009. The microbiological limits established by ISO 17516 are 100 CFU/mL, including for *R. mannitolilytica*. However, it is not possible to directly compare our results to this threshold, as the analysis conditions — including inoculation method, culture medium, incubation temperature and duration — differed between the ISO 17516 standard and our laboratory protocols. These differences may have influenced bacterial enumeration. In the context of an outbreak investigation, the most appropriate conditions to recover the pathogen of interest should be employed. Using patient isolates, we determined that the optimal conditions for growing *R. mannitolilytica* involved the use of a rich medium (Columbia blood agar) and incubation at 37 °C. In contrast, the conditions applied in parallel for water sample testing according to the European Pharmacopoeia (Ph. Eur.) were suboptimal, as *Ralstonia* was not detected in some samples.

The water mist spray is made up of just natural mineral water and nitrogen propellant. Nitrogen could promote the growth of certain commensal bacteria. Interestingly, the plant-pathogenic bacterium *Ralstonia solanacearum* uses inorganic nitrogen metabolism for virulence, ATP production and detoxification [31]. However, we did not find any data concerning *R. mannitolilytica* and nitrogen.

Contamination of commercial water sprays by *Ralstonia* and *Burkholderia* spp. has previously been documented in France, leading to the withdrawal of certain batches by national health authorities [32,33]. We have found no reports regarding the withdrawal of similar products in other countries. This study presents the first documented nosocomial outbreak associated with this type of product.

Given the infectious potential of *R. mannitolilytica* in the specific hospital populations and its resistance to antimicrobial agents, the risk–benefit balance must be carefully evaluated. In our hospital, these sprays had been used for several years for patients’ refreshment, particularly during heatwaves, and oral care in cases of severe mucosal dryness or mucositis, providing considerable comfort according to healthcare staff. Nevertheless, the IPC unit had recommended that these sprays should not be used in units with high-risk patients, such as the ICU and oncology units. They should be reserved for situations where the comfort benefits outweigh the microbiological risks. In view of the current outbreak, our hospital has decided to permanently withdraw this type of product throughout the institution, regardless of the brand. As an alternative, we proposed the use of single-use wipes soaked in chilled bottled water.

Water sprays are not the first cosmetic products to be linked to outbreaks in healthcare environments. Notably, outbreaks linked to liquid soaps contaminated with *Pseudomonas aeruginosa* [34] and pre-moistened ‘Sinaqua’ wipes contaminated with *Burkholderia cepacia* complex [35] illustrate how such products, often perceived as low-risk, can pose a substantial threat to vulnerable patients, especially immunocompromised patients. These events highlight the need for a high level of vigilance, prompt investigations in case of clusters, and a careful risk–benefit assessment when using non-sterile or borderline medical-cosmetic products. Decisions should balance regulation vs restriction or even prohibition in high-risk units. Infection prevention and control teams play a central role in evaluating these risks, guiding product selection and supporting safe practice implementation.

Concerning communication outside our hospital, the Cantonal Medical Office ensured prompt notification to other healthcare facilities and issued an intercantonal notification, leading to a coordinated response. The medical device vigilance officer played a crucial role in directing the report to the appropriate federal office (FSVO rather than Swissmedic), as the water sprays are classified as cosmetics. The FSVO then took responsibility for exchanges with the manufacturer and for reporting the contamination of the product. This underscores the importance of a collaborative system and global monitoring, which enabled an effective response.

We report here a small outbreak of *R. mannitolilytica* including three patients in our hospital and a possible fourth patient in another hospital. None of these patients had a clinical infection, only colonisation. However, one patient experienced central line contamination, which could potentially have led to a severe infection. No definitive epidemiological link could be established with the fourth patient, hospitalised in another hospital, as no remaining sprays from the contaminated batches were available there for analysis. Nevertheless, whole genome sequencing revealed that the isolate identified in this patient was indistinguishable from those found within our institution, which strongly suggests exposure to a common source. 

## Conclusion

This small outbreak of *R. mannitolilytica* led to the identification of a contaminated cosmetic product, routinely used in patient care, as a source of nosocomial infection, prompting a reconsideration of its widespread use in hospital settings. Effective and centralised communication was crucial for coordinating both messages and actions.

## Data Availability

Genomic data are publicly available in DDBJ/ENA/GenBank under BioProject no. PRJEB82839 [29].
